# Supplementary material for: A Comparison of Cats (Felis silvestris catus) Housed in Groups and Single Cages at a Shelter: A Retrospective Matched Cohort Study
Source: Animals (Basel). 2018 Feb 14;8(2):29. doi: 10.3390/ani8020029 (PMC5836037; doi:10.3390/ani8020029)
Supplement: Supplementary file 1 [file animals-08-00029-s001.pdf]

animals

MDPI

Article

A Comparison of Cats (*Felis silvestris catus*) Housed in Groups and Single Cages at a Shelter: A Retrospective Matched Cohort Study

Malini Suchak * and Jacalyn Lamica

Table S1. Spearman's rank correlations between dependent variables. Note that only cats who were released live could be returned, therefore the correlation between those two variables is not present. Significant correlations are in bold. ***denotes p<0.001, ** p <0.01, *p<0.05

	Time on adoption floor	Moves to isolation	Moves	Average days/loca tion	Bite cases	Offsite moves	Live release	Return
LoS	**−0.28*** **	**0.33*** **	**0.66*** **	**0.60*** **	0.10	**0.37*** **	−0.11	0.06
Time on adoption floor		**−0.43** **	**−0.45*** **	0.06	−0.10*	−0.09	0.04	0.05
Moves to Isolation			**0.51*** **	**−0.141* **	−0.05	**0.21** **	−0.02	0.02
Moves				−0.14	0.09	**0.56*** **	−0.04	0.09
Average days/loca tin					0.02	−0.04	−0.09	−0.03
Bite cases						−0.05	−0.09	−0.14
Offsite moves							−0.07	0.01
